# Hard real-time closed-loop electrophysiology with the Real-Time eXperiment Interface (RTXI)

**DOI:** 10.1371/journal.pcbi.1005430

**Published:** 2017-05-30

**Authors:** Yogi A. Patel, Ansel George, Alan D. Dorval, John A. White, David J. Christini, Robert J. Butera

**Affiliations:** 1 Bioengineering Graduate Program, Georgia Institute of Technology, Atlanta, Georgia, United States of America; 2 Department of Biomedical Engineering, Georgia Institute of Technology and Emory University, Atlanta, Georgia, United States of America; 3 Department of Physiology and Biophysics, Weill Cornell Medical College, New York, New York, United States of America; 4 Department of Bioengineering, University of Utah, Salt Lake City, Utah, United States of America; 5 Department of Biomedical Engineering, Boston University, Boston, Massachusetts, United States of America; 6 School of Electrical and Computer Engineering, Georgia Institute of Technology, Atlanta, Georgia, United States of America; Universite de Montreal, CANADA

## Abstract

The ability to experimentally perturb biological systems has traditionally been limited to static pre-programmed or operator-controlled protocols. In contrast, real-time control allows dynamic probing of biological systems with perturbations that are computed on-the-fly during experimentation. Real-time control applications for biological research are available; however, these systems are costly and often restrict the flexibility and customization of experimental protocols. The Real-Time eXperiment Interface (RTXI) is an open source software platform for achieving hard real-time data acquisition and closed-loop control in biological experiments while retaining the flexibility needed for experimental settings. RTXI has enabled users to implement complex custom closed-loop protocols in single cell, cell network, animal, and human electrophysiology studies. RTXI is also used as a free and open source, customizable electrophysiology platform in open-loop studies requiring online data acquisition, processing, and visualization. RTXI is easy to install, can be used with an extensive range of external experimentation and data acquisition hardware, and includes standard modules for implementing common electrophysiology protocols.

This is a *PLOS Computational Biology* Software paper.

## Introduction

Advances in stimulation (electrical, optical, biochemical) and measurement (electrical, biochemical, optical) techniques have increased the spatial and temporal resolution with which researchers can monitor or perturb biological activity. Using such tools and techniques in a closed-loop paradigm, where an acquired signal is used to compute the system output, can enable observation of physiological function, development and validation of computational models, as well as investigation of causal relationships in biological systems. This requires closed-loop control that operates on timescales that are physiologically relevant, which span tens of microseconds at the ion channel level to minutes at the behavioral level. Furthermore, the closed-loop control needs to be hard real-time (RT)—operating on a strict schedule for acquisition, processing, and yielding a computationally determined output with guaranteed performance bounds appropriate for the timescales of interest. Such hard RT, closed-loop control is implemented in industrial applications (e.g, aerospace, robotics, stock markets); however its use in biological research is stymied due to the relative high cost of commercial systems, and lack of flexibility in customizing closed-loop protocols, performance, and features.

**Fig 1:**
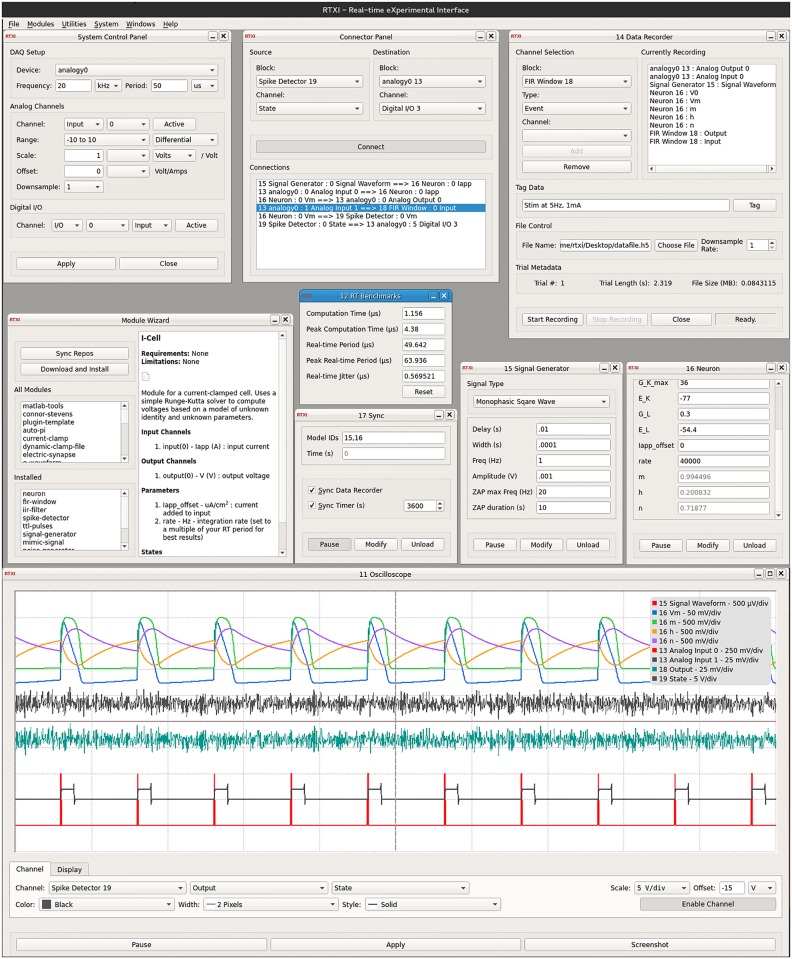
Screenshot of arbitrary RTXI workspace with core and custom modules. The workspace is intended to demonstrate the level of user-configurability provided by RTXI, such as channel configurations, combinations of data I/O connections, and saving virtually every programmed element of each module. Additional custom modules are shown (e.g., Spike Detector, FIR Filter, Signal Generator, Neuron Model, etc) to demonstrate the versatility of RTXI’s API system for creation of any utility or model to be used in hard RT, closed-loop experiments. We refer the reader to the up-to-date user manual (S3 File) and tutorials (S3 File) available online for more information on how to navigate the RTXI workspace.

The Real-Time Experiment Interface (RTXI, http://www.rtxi.org, Fig 1) is an open source software platform for hard RT, closed-loop data acquisition (DAQ) and experimental control in biological experiments used by over 70 labs worldwide. This manuscript provides a technical and practical overview of RTXI’s architecture and features, as well as highlights select novel applications. Functionality of RTXI’s architecture and features has been validated both computationally through load testing and performance characterization, and experimentally in multiple setups of different biological systems and varying time scales.

RTXI is based on Xenomai, a Real-Time Linux framework [1, 2] and can be run or installed on any desktop PC by using the Live CD or by manually compiling the source code. RTXI can interface with an extensive range of external experimentation and data acquisition hardware, and includes standard modules for implementing commonly used electrophysiology protocols. Modules contain function-specific code that can be used in combinations to build custom workflows, experimental protocols, and interfaces, thereby eliminating the need to code all aspects of each experiment protocol from scratch. The power, flexibility, and stability of RTXI has made it possible for users to implement complex custom closed-loop protocols in a variety of cardiac [3–7] and neuronal systems at the single cell [8, 9], cell network [10], animal, and human electrophysiology [11] levels. Dozens of publications (S3 File) have used RTXI. Examples include investigation of the contribution of specific ion channels or synaptic receptors to spiking and bursting activity in a variety of neuronal cell types, oscillatory behavior of pacemaker neurons [12], the effect of network topology and intrinsic neuronal properties on population activity in a hybrid network [13], and effects of transcranial alternating current stimulation (tACS) on cortical activity [11, 14]. Each example utilized RTXI to create a custom, hard RT closed-loop protocol with the goal of dynamically probing the target system.

## Design and implementation of the Real-Time eXperiment Interface

### System architecture

An RT control system for closed-loop control typically runs in an iterative computational loop with the smallest possible nominal cycle period, a minimum amount of cycle-to-cycle variation (jitter) in the actual period, and the shortest possible system latency (delay from input to computed output) determined by the specific application [15]. Standard desktop computer operating systems, including Linux, are built upon a monolithic kernel whose scheduler is engineered to balance distribution of resources amongst the various active threads and respond to hardware and software triggered interrupts in a resource-efficient manner. This results in standard desktop operating systems providing only “soft” RT performance. In soft RT systems, occurrence and timing of data acquisition, processing, and output generation events is neither guaranteed nor bounded. A lack of such guarantees can lead to substantial, yet often unnoticed, effects in experimental control [16–18]. For example, in the case of dynamic clamp [19–22], a soft RT system may occasionally wait so long to compute the injected current that the actual value of the membrane potential has changed significantly in the meantime. The resulting experimental dynamics may look acceptable but still be wrong, since they are based on incorrect assumptions about the state of the cell. In practice, this means that phase-dependent stimulation may be delayed or occur at the incorrect physiologically-relevant time.

To achieve hard real-time performance, RTXI uses a Real-Time Operating System (RTOS). An RTOS enables hard RT performance by modifying the operating system’s native kernel architecture to enable priority-based pre-emption of processes, allocation of memory, and communication with on-board hardware for data and file I/O. To enable hard RT control, RTXI uses Xenomai, a real-time framework, which installs a micro-kernel alongside the standard Linux kernel. The micro-kernel consists of its own scheduler and interrupt handler and places the standard Linux kernel in a low-priority state, allowing dedicated processes to be prioritized. For example, Xenomai’s prioritization of RTXI enables hard RT control of periodic tasks such as sampling from experimental equipment, performing computations, and generating output signals. The use of an RTOS also minimizes system and hardware latencies, resulting in faster sampling rates, computation times, and file I/O. For some experimental designs with closed-loop feedback, a higher sampling rate also improves the stability of the protocol [23].

### Application architecture

**Fig 2:**
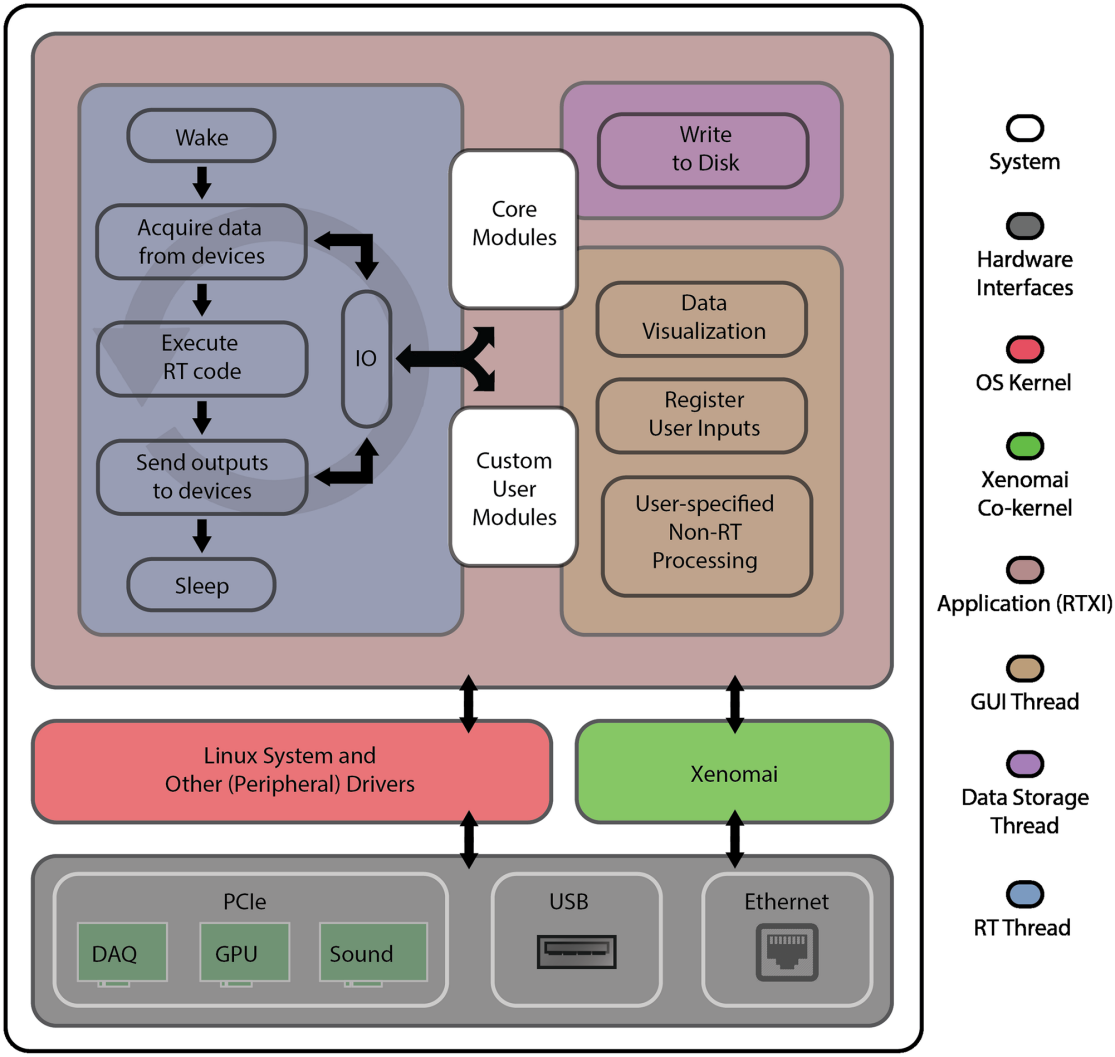
System architecture. The bottom block depicts the hardware layer with which RTXI interfaces. RTXI is capable of interfacing with DAQs using either PCI/PCIe, USB, or Ethernet interfaces (see Compatible hardware for more information). Hard RT communication with hardware devices is achieved through Analogy, a set of drivers within the Xenomai framework. The top block of the diagram illustrates the core architecture of RTXI. On each cycle of the RT period, the RT Thread wakes up, acquires new data, executes instructions defined within the hard RT function of both core and user modules (see Custom modules and Application Programming Interface (API)), outputs data to the DAQ, and returns to sleep (idle). Transmission of data to/from different modules is handled by the IO class. When the RT Thread is idle, resources are made available to other system applications and functions. The GUI and Data Storage Threads continuously run with a static period to provide a stable balance between hard RT performance and handling of user input, updating of visualizations, and data storage.

RTXI is written in C/C++ and utilizes three threads to process: 1) hard RT data acquisition and experimental control, 2) user interactions, and 3) data storage. Fig 2 provides an overview of the complete system architecture. The RT thread is instantiated with the highest system priority and controlled by the micro-kernel. The second user interface and experience (UI/UX) thread, powered by the Qt [24] and Qwt [25] graphical user interface (GUI) frameworks, processes user inputs to RTXI and online data visualization in soft RT. The final soft RT thread continuously reads and writes data to disk. With the advent of multi-core desktop computers, users are also able to instantiate additional soft RT threads for online data processing. All threads run in the same process address space, making it easy to share data for processing, updating visualizations, and data storage. RTXI’s architecture allows it to be used as an open-loop experimental control and data acquisition system [26–29], or even as a simulation environment.

The RT thread wakes on each clock cycle and executes all DAQ operations, module functions, and RT system functions. Module functions refer to both base system operations, such as transmitting data across modules and writing data to system buffers, and all RT operations implemented within custom modules. During this step, modules can post RT events—which notify other modules about state changes and user-initiated events, e.g., unloading or loading of new modules—to queue. These events are then executed by the RT thread once all module computations are complete.

An important advantage of this modular application architecture is the ability to load and unload both core system and user-created modules, change parameters, and modify any system variable online without halting closed-loop execution or affecting hard RT performance. Commercial systems, such as Simulink (MathWorks, Natick, MA), Signal (Cambridge Electronic Design, Cambridge, England), LabVIEW (National Instruments, Austin, TX), and Tucker David Technologies (Alachua, FL) typically require halting execution and recompiling the modified loop prior to being able to continue execution. Open-source platforms, such as NeuroRighter [29], MANTA [30], PLDAPS [31], and the Open Ephys GUI [32], provide closed-loop control with varying degrees of flexibility, but none enable hard RT closed-loop control. Such limitations are not ideal for investigation of causal relationships in biological systems.

RTXI enables users to create custom real-time algorithms and protocols for closed-loop processing and visualization. Debugging custom algorithms and protocols can be difficult in a hard real-time environment, especially without expertise or knowledge on how to probe the Linux kernel during execution. To ease the process of developing hard real-time algorithms and protocols, RTXI can be easily configured to run in non-real-time (non-RT) mode with debugging support. This configuration option is provided by the RTXI installation scripts and is outlined on the RTXI website (S3 File). When run in non-RT mode, users can obtain a tracelog of events to identify where and why the system crashed, occurrence of race conditions, and system resource usage.

### Compatible hardware

RTXI interfaces with experiments through a variety of hardware interfaces, including PCI/PCIe based DAQs from National Instruments and Sensoray, Ethernet based devices such as cameras and commercial amplifiers, as well as USB-based acquisition devices (S3 File). The hardware used with RTXI should be chosen based upon the hard RT needs of the custom protocol. Devices interfacing through the PCI/PCIe and Ethernet interfaces are capable of providing hard RT closed-loop performance with the appropriate drivers. PCI/PCIe hardware can achieve sub-millisecond latencies, while Ethernet devices can provide millisecond latencies. USB devices can provide closed-loop functionality with the appropriate driver, but the non-deterministic bounds of the USB protocol prevent hard RT control.

### Portability and sharing

RTXI allows users to move developed and tested modules, algorithms, and entire closed-loop protocols from one computer to another without significant overhead. Once the workflow and protocol have been set up, the entire workspace can be saved to an XML-based workspace settings file. This file saves all core system specifications set by the user, loaded modules along with their parameter and state values, and connections between modules. The settings file can then be used on any other computer with RTXI and the appropriate modules installed to restore the workspace. Furthermore, existing workspace settings files can be used as a starting point for creating new experimental protocols. This reduces the chances of errors when setting up a complex protocol with many modules and provides an easy mechanism for sharing custom protocols. Users can share all elements of their custom protocols—modules, settings files, screenshots, data, etc—on RTXI’s GitHub page (S3 File).

### Core modules

RTXI’s base functionality (data acquisition, processing, and visualization) is achieved through core modules designed to provide hard RT closed-loop performance. A set of core modules with minimal computational overhead are compiled as shared object libraries during the RTXI installation process and linked to RTXI at run-time. This approach leads to lower system overhead and thus greater flexibility for users to define custom protocols that may require greater computational resources.

#### System control panel

**Fig 3:**
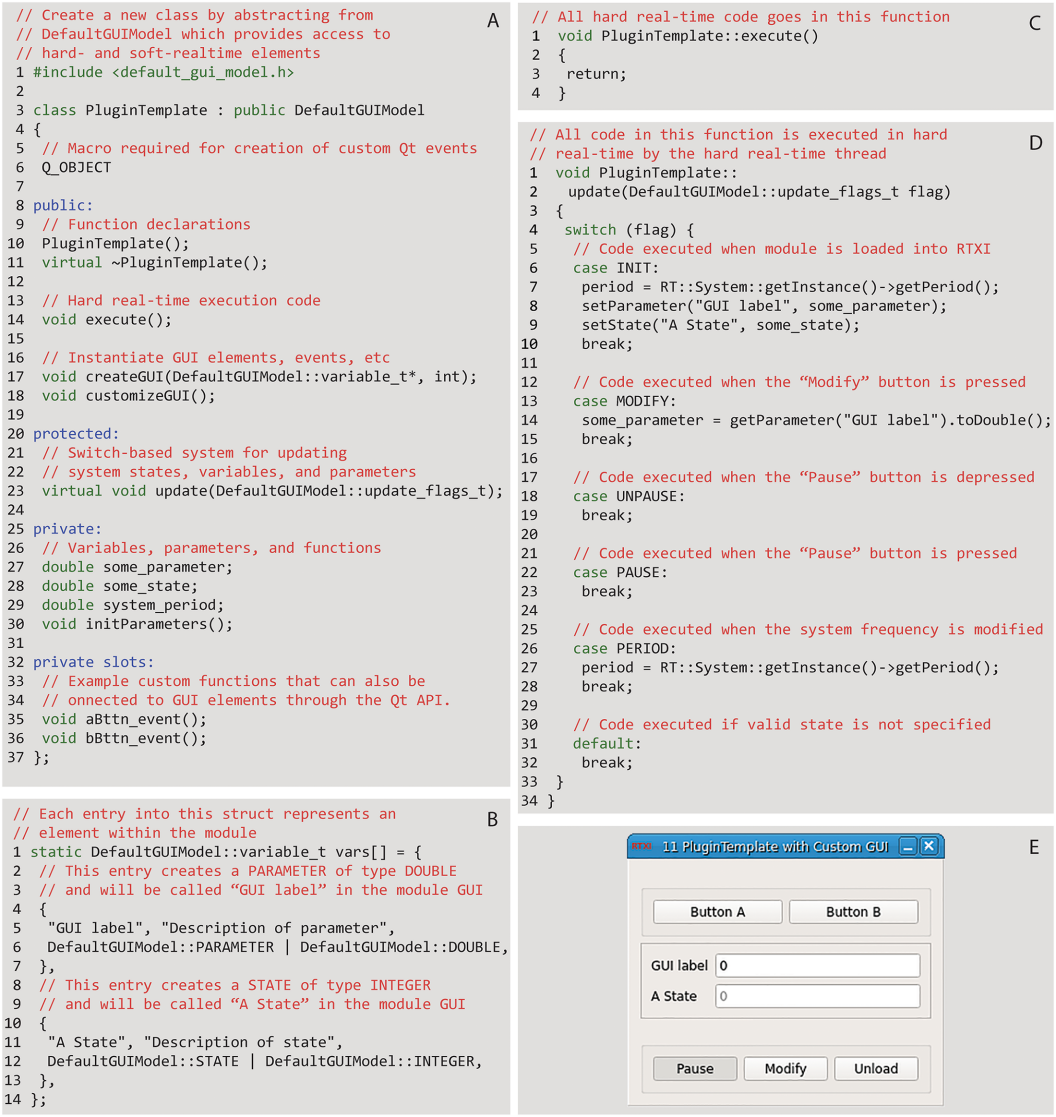
Example template code available (S3 File) to users for writing custom modules, and corresponding module loaded within RTXI. (A) Basic C++ header (plugin_template.h) for an RTXI module. (B) Users can declare inputs, outputs, states, parameters, and events for custom modules through the DefaultGUIModel struct and (C) implement hard RT code within the execute function. (D) The update function allows execution of state-specific code for the module. (E) The module after it is compiled with the provided Makefile and loaded into RTXI. Each element of the module GUI is tied to a specific line of code. For example, when “Button A” is clicked, the aBttn_event() (line 35, A) code is executed. Similarly, the “GUI label” (lines 5–6, B) and “A State” (lines 11–13, B) components of the GUI are created by the vars struct and their values are initialized from the INIT block of the update() function (lines 8–9, C). Visit the GitHub repository (S3 File) for complete corresponding C++ implementation (plugin_template.cpp) file.

The System Control Panel shown in Fig 1 is the primary interface for configuring RT system settings and DAQ I/O channels. The detected on-board DAQs are listed in the Devices drop-down menu (e.g., analogy0). Users can set the Frequency or Period of the RT system, which is used as the data acquisition (I/O) rate and determines the computation time available per cycle. All available AI, AO, and DIO channels are automatically detected from the installed DAQ and listed for the user to enable and configure. For example, users can configure the measurement mode (Ground-referenced, Differential, etc), scale the input measurements to account for hardware gains, change the measurement range, and add or remove offsets to each channel individually. Users can also configure available Digital I/O channels through this panel. When the channel configurations are “applied”, an event is registered on the system stack, notifying all open modules of the changes. Users can respond to these changes through the update function of their custom modules (see Fig 3D).

#### Data recorder and HDF5 files

The Data Recorder module shown in Fig 1 allows saving of virtually anything within RTXI. Users first select the module of interest, and then have the option of saving any module element specified within the DefaultGUIModel::vars struct (see Fig 3B). Parameters for all modules are automatically registered at the beginning of a recording session. If modified during execution, the updated value is registered to the data file with a timestamp. Users can also “tag” various time points throughout their experiment to note relevant events. Events are timestamped and registered as a list of tags within the specified data file. A global downsampling option is available to reduce output file sizes. In between active recording sessions, the size and number of trials present in the specified data file, and length of the previous trial are displayed in the metadata section. If a user chooses an existing file to write data to, the option is presented to either overwrite or append the data file.

Experiment data and metadata saved by the Data Recorder are stored in the Hierarchical Data Format (HDF5). HDF5 [33] is an open data model that is increasingly popular for representing large and complex data, data relationships, and their associated metadata. HDF5 file read and write operations are supported by many common analysis frameworks and languages (e.g., MATLAB, Python, Julia, R, etc). Acquired raw and processed data, module states and parameters, system configurations, and almost any other value can be synchronously saved to a single HDF5 file by simply selecting the appropriate signal and adding it to the list of active recording channels. In addition, any computed value or intermediate signal can easily be captured for offline debugging or validation of RT algorithms and processing. When a parameter value is modified on-the-fly during data acquisition, the new value is automatically time stamped and stored into the data file. The Data Recorder also includes the ability to timestamp data with tags to experimental events or making notes and includes DAQ channel configuration details for all active channels. For precise control of data recorder start and stop times, the Data Recorder can easily be coupled with the Sync module, shown in Fig 1, which can be used to control the state of numerous modules and the data recorder all at once.

**Fig 4:**
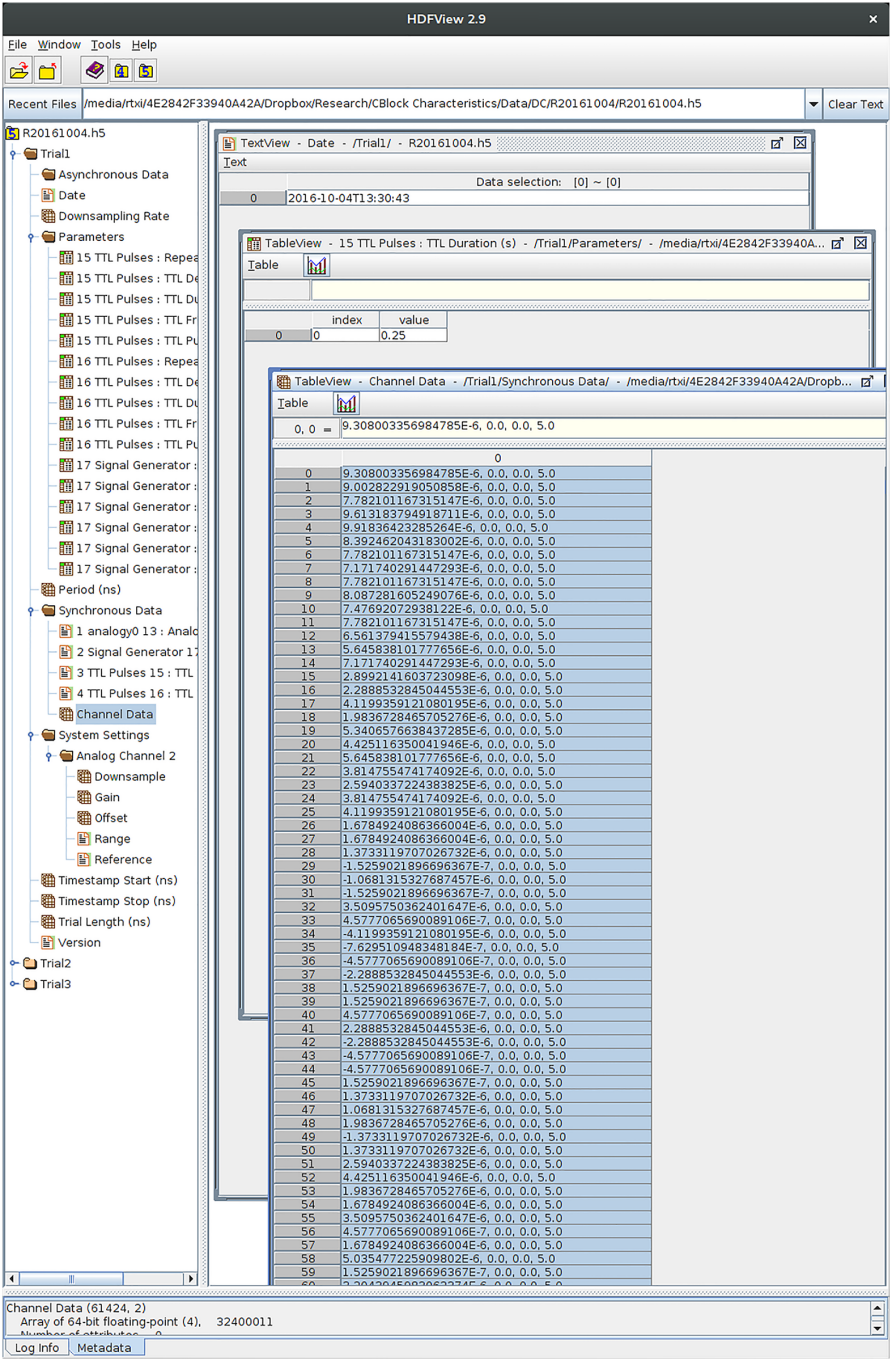
Hierarchical data format data file generated by RTXI. Each trial is represented as an ordered group of data and metadata objects. By default, RTXI saves parameter values from all modules when recording is started into the “Parameters” group. All enabled DAQ AI channel configuration settings are also saved into the “System Settings” group. HDF5 files can be opened, modified, and appended to from a variety of post-hoc analysis frameworks (e.g., HDFView, MATLAB, R, Python).

Fig 4 depicts the structure of an RTXI-generated HDF5 file. Scripts and analysis tools are provided online for importing RTXI-generated HDF5 files into a single MATLAB structure for post-hoc analysis. RTXI-generated HDF5 files are compatible with many commercial and free software applications for a variety of platforms. There is no required proprietary software for viewing or analyzing data stored in RTXI-generated HDF5 files. Much of the available software also supports editing data in place within the HDF5 file or appending new data to an existing file. This allows users to add associated data such as images, post-processed data, or additional notes.

#### Connector

Passing of data between core and custom modules within the RTXI workspace takes place through the Connector module shown in Fig 1. The module’s simple interface allows mapping of outputs from a module or DAQ AI channel to the input of another module in various configurations (one-to-one, one-to-many, many-to-one, etc). The specific input and output signals available to a module are defined in the module’s code (see Fig 3). In cases where a many-to-one connection is formed, the inputs are summed prior to passing the data to the specified input. For example, Fig 1 shows the data from DAQ AI channel 0 and the output of the Signal Generator module connected to the input of the Neuron module. Simultaneously, the output of the Neuron module is sent to the Spike Detector module as well as out to hardware via DAQ AO channel 0.

#### Oscilloscope

RTXI’s built-in real-time, high-speed, fully configurable Oscilloscope shown in Fig 1 module allows visualization of signals, parameters, states, and events from any loaded core or custom module. Similar to the Data Recorder and other system modules, the Oscilloscope can access data of any kind from any loaded module within the workspace. To visualize the signal, the user selects the block of interest on the “Channel” tab, which provides a list of available streams along with options to customize the color, style, and vertical scale (per division) of the selected signal. Once the modifications are “applied”, the enabled signal is visualized with the configured settings and a legend entry is created listing the enabled channels by name along with their respective vertical scale. A second tab, titled “Display” allows configuration of the time scale (per division), the Oscilloscope’s refresh rate, and specify trigger settings. Screenshots can be saved online during experiments for quick figure generation.

#### Real-time benchmarks

Online evaluation of system performance is useful when designing RT algorithms, debugging experimental setups, and even during experiments. The RT Benchmarks module shown in Fig 1 (pane with blue top bar) provides key statistics about the RT performance of the system—including the computational time, RT period, and the RT period’s jitter—by using timestamps directly from the real-time system’s clock. These benchmarks have been shown to be critical in evaluating hard real-time performance [18]. The computational time is inclusive of all system and custom modules loaded into the workspace, enabling evaluation of the full hard RT closed-loop execution time of each custom protocol. The RT period and RT period’s jitter provide a means for viewing the actual period of the closed-loop system. The RT period should never exceed twice the desired RT period, otherwise hard RT behavior is compromised. Furthermore, each statistic reported by the RT Benchmarks module can be saved to an HDF5 file through the Data Recorder for post-hoc validation and verification of hard RT performance during the experiment. As described in the RTXI documentation (S3 File), end users are strongly advised to benchmark their hardware and modules to ensure that the performance they require is actually being delivered.

#### Module wizard

There are over 50 modules available to RTXI users through the RTXI GitHub repositories (S3 File). The Module Wizard shown in Fig 1 was created to ease the process of downloading, installing, and updating new and existing modules. Users can quickly synchronize with the GitHub repositories and view details about each module, including the specific functions, how it works, and its development status. If a specific module is of interest, the user can download and install the module directly from within the Module Wizard and within seconds load the new module into their workspace.

#### User preferences

The User Preferences module shown in Fig 1 enables customization of default file locations for various file I/O operations. Users can specify folder locations for workspace setfiles and HDF5 data files. In addition, users can specify buffer sizes for the Data Recorder, which can be beneficial when acquiring large amounts of data at high sampling rates. All settings specified within the User Preferences module are set as the default values and saved to the global RTXI configuration file.

### Custom modules and Application Programming Interface (API)

One core strength of RTXI is the ability to create custom RT modules without being restricted to hardware and software-defined boundaries, which is a common obstacle with commercial systems. RTXI users implement custom experimental protocols by writing all aspects of their RT protocol in C++-based modules. The use of C++ as the language of choice allows users to incorporate a variety of established libraries, such as LAPACK, Boost, and GNU Scientific Library for processing and data visualization, while capitalizing upon the strengths of C++ such as recursion, object-oriented programming, and abstraction. This system for creating custom closed-loop protocols enables complete freedom with respect to customization of closed-loop RT protocols without any virtual limit on what can be implemented.

To get started, users are encouraged to reference the Plugin Template, which provides the critical elements required for creating a custom RTXI module. Fig 3 provides an overview of the Plugin Template files and code. The Plugin Template files include a Makefile, which is used to define compilation rules on how files should be compiled, which external libraries are to be included, and how to package the individual pieces together to form a shared library object that is RTXI-compatible. In addition, one header (plugin_template.h, Fig 3A) and implementation (plugin_template.cpp) file are included as examples that can be built upon.

All custom modules are abstracted from the DefaultGUIModel class, which includes all elements necessary for interfacing with both the RT and GUI threads. When creating a new module, users create the various I/O elements for that module through the vars structure (Fig 3B). To run specific code in RT, the code is placed in the execute() function (Fig 3C), which is common to all system and custom modules and processed at each time step. Functions called from within the execute function are also run in RT, while all code that is not in or called by the DefaultGUIModel::execute function is run on the soft RT GUI thread. This enables a simple mechanism for separating hard RT and soft RT tasks without significant programming overhead for the end user.

Users are able to customize the GUI of the module by including a variety of labels for model parameter and state variables, buttons and other elements for module control, as well as plotting elements for online analysis and visualization through the customizeGUI() function. By default, each module consists of three automatically generated buttons for starting or pausing execution, updating user-set parameters, and unloading itself from the workspace. Customization of the GUI is achieved by using the Qt framework, which provides a variety of GUI elements such as buttons, lists, text boxes, etc. Users can include signals and slots, which is a mechanism for calling specific functions based upon the occurrence of an event (e.g., button press → meet threshold criteria → specific animal behavior). For example, users can include a radio button in their module that has a signal-slot connection to a plot. When a user clicks the radio button, a signal is emitted and tells the module to display a scatter plot of data, which is otherwise hidden. The same signal-slot mechanism can be used to create buttons that drive outputs via the DAQ, call specific protocol functions, or generate notifications when an event occurs. Users are also encouraged to check the listing of available modules on the RTXI GitHub page for examples or potentially suitable modules that can easily be adapted to meet individual needs.

Updates to module variables, responding to changes in RT system settings applied through the system control panel, and controlling the state of each module takes place through the update() function (Fig 3D). Each module can be in one of five states—which are responsible for initializing all module elements (INIT), registering UI/UX commands to the module (MODIFY), responding to changes in the RT system frequency (PERIOD), and controlling whether or not the module is running (UNPAUSED) or not (PAUSED). Users can customize any state for experiment-specific needs.

## Results and discussion

### System characterization and load testing

**Fig 5:**
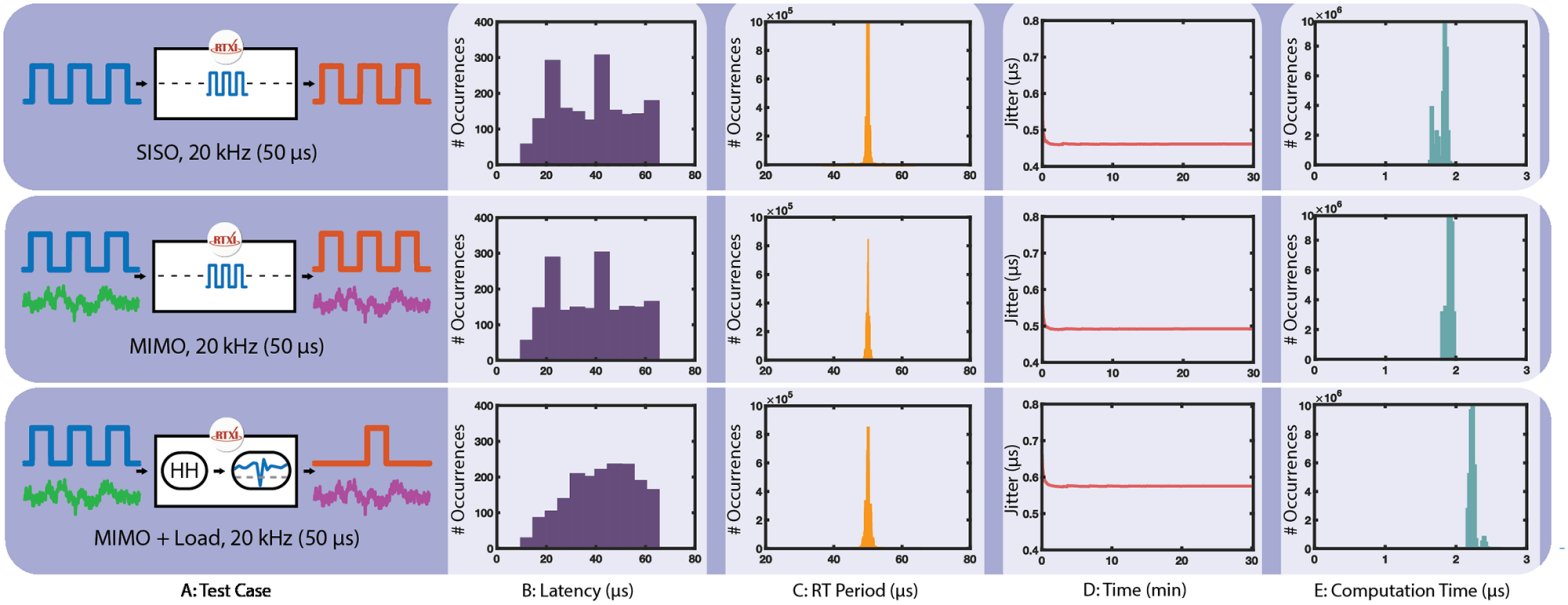
System performance under different computational loads and closed-loop configurations. (A) System configurations tested. (B) System I/O latency does not exceed twice the system period (50 *μ*s) nor are I/O events missed in all test cases, demonstrating hard RT performance and a delay of no more than one sample. (C) Distribution of measured RT periods in each tests case by the Real-Time Benchmarks module. The RT period should never exceed twice the desired RT period, otherwise hard RT behavior is compromised. The increase in RT period variability with increasing computational loads is predominantly from the initial time frame after which a protocol starts execution. This is represented by the RT period jitter data (D), which demonstrates the RT period stabilizing within the first few minutes of execution. (E) Time spent processing the execute() function of all loaded module on each RT loop cycle.

We evaluated RTXI’s performance in different I/O configurations and computational loads (Fig 5). Tests were performed by providing a randomly triggered input signal (square wave, 50% duty cycle, 5 Vpp) to one of the DAQ AI channels. Each test case was run for 30 minutes (1800 total input events). In Case 1 (top), 1 AI channel was used to measure the input signal, and 1 AO channel was used to output the same signal, with no processing in the loop to evaluate system performance in a single-input, single-output (SISO) configuration. Case 2 (middle) used the same configuration as Case 1, but included 2 AI and 2 AO channels. Only one AI channel received the input signal. The other AI channel was left floating and directly connected to the second AO channel, allowing characterization of system performance in a multi-input, multi-output (MIMO) configuration. Case 3 used the same configuration as Case 2, but with added processing within the loop. The Hodgkin-Huxley Neuron model was configured to receive the test input signal and the output of the Neuron model (membrane potential, V_*m*_) was connected to the Spike Detector module. The Spike Detector module determines if an action potential has occurred by checking for a threshold-crossing event. When an action potential is detected, the Spike Detector outputs a TTL pulse that is then sent out via the AO channel.

A separate data acquisition system (MATLAB, NI USB-6341) was used to sample (250 kHz) the AI and AO signals from each case to quantify the I/O latency with each configuration. The RT period, RT period jitter, and computation time are measured with the Real-Time Benchmarks module and recorded with the Data Recorder. All tests were performed with an RTXI (v2.1) system frequency of 20 kHz on a desktop computer with an Intel Core i5 Quad Core Processor (3.40 GHz), 32GB of physical RAM, 10,000 RPM hard drive, Radeon HD 8570 graphics card, and an NI PCIe-6259 DAQ. All system and application daemons were killed prior to performance evaluation (recommended) because such background processes can prevent resource allocation or occupy significant amounts of CPU time. All analysis was performed using MATLAB 2016 (MathWorks, Natick, MA, USA). Data shown in Fig 5 is from one RTXI installation, however additional performance characterization results are available online from different installations (S3 File). Each test case demonstrates I/O processing of 1800 events with the desired RT period, demonstrating hard RT performance and a delay of no more than one sample.

Fig 5A summarizes each case scenario used to characterize system performance. With a system frequency of 20 kHz, the system has 50 μs to complete input, processing, and output events. Histograms of latencies measured by an external data acquisition system are shown in Fig 5B. For all test cases, the system latencies are distributed around 50*μ*s. If a latency greater than 100*μ*s had been measured (twice the system period), the hard aspect of the real-time system would be lost due to the system missing deadlines for events (e.g., input not acquired, output not generated).

Fig 5C–5E of show system performance metrics measured and reported by the core RT Benchmarks module. Fig 5C demonstrates that the measured RT period is unimodal and tightly distributed around 50*μ*s in all I/O configurations and system loads. As the system load increases, the variance of the RT period increases, but the mean stays closely centered at 50*μ*s. The RT period’s jitter is shown in Fig 5D and is consistent throughout RT execution, demonstrating that RTXI has access to sufficient resources, is prioritized by the real-time scheduler, and can provide the requested performance. If RTXI was unable to meet the performance demands of the configuration, the RT period, and thus the jitter, would vary significantly over the course of an experiment. The final column of Fig 5 represents the time taken to complete the input, processing, and output events. As the system configuration and load increase, the computation time also increases but without losing hard real-time performance (as shown by Fig 5C–5D).

### Use cases

#### Use case 1: Replication and suppression of learning-induced membrane and synaptic plasticity

RTXI’s most common application is for the dynamic-clamp technique (of the many examples, some include: [20–22, 34]). This has led to significant advances in fundamental concepts in neuroscience and related fields.

Feeding behavior in the mollusk Aplysia is modified by various forms of associative learning, including classical and operant conditioning, and alter the central decision-making processes related to feeding actions. Experimental modification of intrinsic excitability and electrical synapses of neurons in the Aplysia feeding central pattern-generating network have been shown to correlate with compulsive-like motor output expressions induced by *in vivo* operant conditioning. Despite this correlation between plasticity and operant conditioning-induced changes, a causal relationship was not shown.

**Fig 6:**
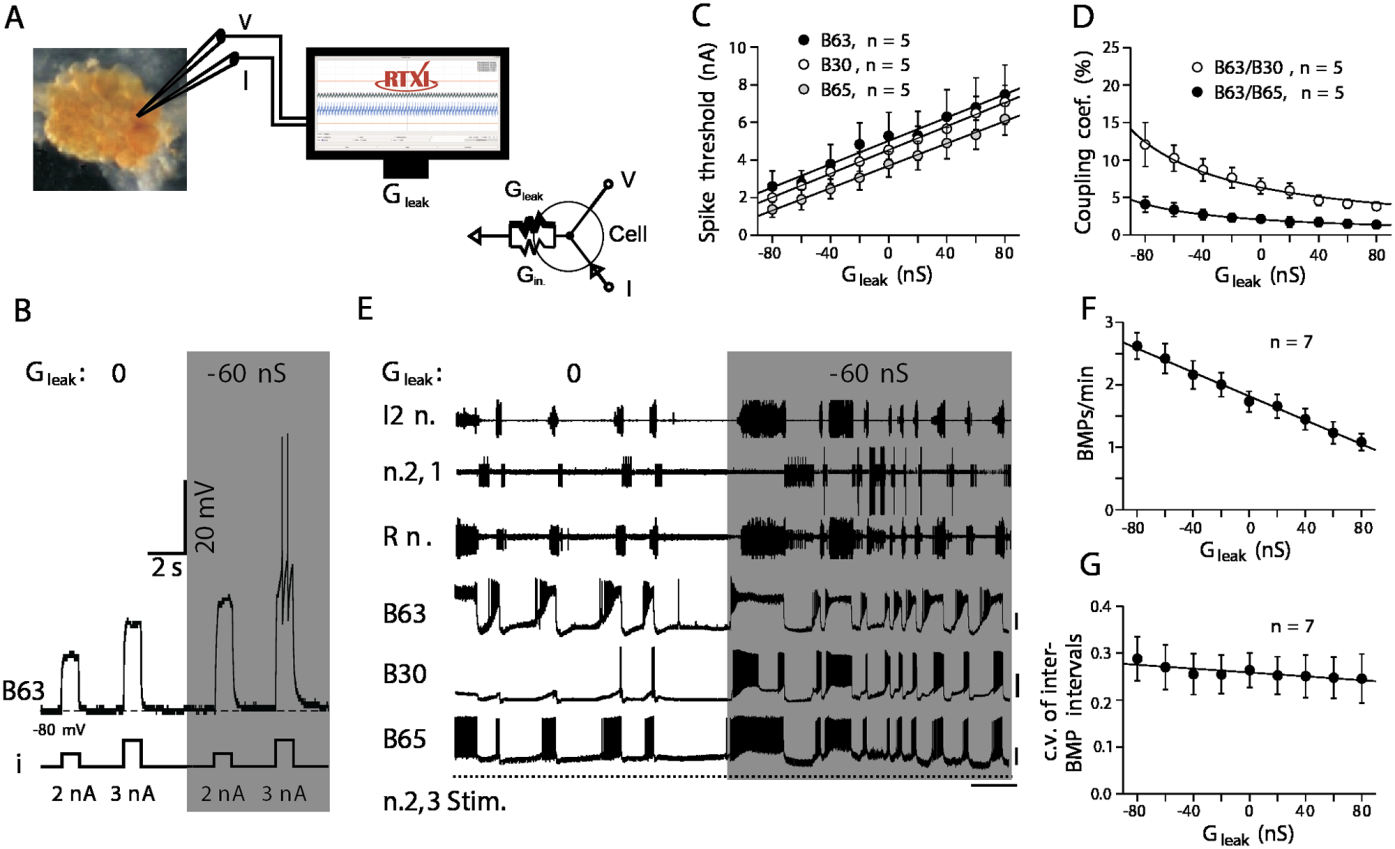
Artificially modulating neuronal excitability mimics learning-induced increase in frequency, but not the regularity, of buccal motor pattern (BMP) genesis. (A) Experimental protocol and equivalent electrical circuit for the addition of a dynamic-clamp-defined leak conductance (G_*leak*_) to the natural input conductance (G_*in*_) of an individual neuron using RTXI. (B) Introduction of an artificial G_*leak*_ of −60 nS (shaded panel) increased the excitability of a target B63 neuron (indicated by a decrease in spike threshold) compared with that arising from the natural leak conductance alone (i.e., G_*leak*_: 0 nS). Horizontal and vertical scale bars represent 2 s and 20 mV, respectively. In (C), G_*leak*_ was introduced into one of B63, B30, or B65. In (D), G_*leak*_ was introduced into a B63 and current pulses for measuring coupling coefficients were injected into either postjunctional B30 or B65. (For details, see [35] Fig. S1.) (E) In a control preparation, the frequency, but not the regularity, of spontaneous BMP genesis and associated spike bursts in B63/B30/B65 increased in response to a dynamic-clamp-defined G_*leak*_ of −60 nS (shaded panel) introduced simultaneously into the three neurons. Horizontal and vertical scale bars represent 30 s and 25 mV, respectively. (F and G) Quantification of changes in frequency (F), but not irregularity (G), of BMP generation for different values of artificial G_*leak*_ added simultaneously to the three neurons. Group data show means ± SEM and individual sample sizes. All figures obtained and modified with permission from [35].

Using RTXI, Sieling et al [35] used the dynamic-clamp technique, in which simulated membrane and synaptic currents are artificially added or subtracted from neurons, to examine whether selective changes in single conductances governing cell excitability and electrical coupling are responsible for the associative modification of feeding circuit output and behavior (Fig 6). Using *in vitro* preparations of buccal ganglia isolated from naive and operantly trained animals, Sieling et al either enhanced or diminished neuronal excitability and coupling strengths in RT to test for causation and determine respective contributions of synaptic and non-synaptic processes by which associative learning leads to expression of compulsive behavior.

#### Use case 2: Real-time distorted auditory feedback for control of song bird timing

In addition to the widely used dynamic clamp, RTXI’s flexibility and robustness are useful for a variety of investigations testing causal relationships. One such area is Distorted Auditory Feedback (DAF), which is routinely used to assess the effects of auditory input on vocal production and underlying neural activity in songbirds. Some bird species’ songs have been shown to be highly sensitive to acoustic input, showing immediate effects on the timing and acoustic structure of the produced song. Investigation of immediate changes taking place after DAF presentation often requires having a system capable of providing RT acoustic processing and feedback. Commercial systems for RT acoustic processing are available but are expensive and contain a significant number of constraints that must be considered during experimental design. Furthermore, typical experiments using acoustic feedback use detection of a certain template signal, either a certain frequency or more complex combination or sweeps of frequencies, and feedback is delivered upon template detection. Template detection tasks are computationally intensive, requiring spectrogram-based or feature-based techniques [36, 37], comparison across detected elements and templates, and if successful, generate the appropriate feedback signal. These computations have to be done fast enough to enable RT performance while not interfering with other processes such as data acquisition, storage, and visualization.

**Fig 7:**
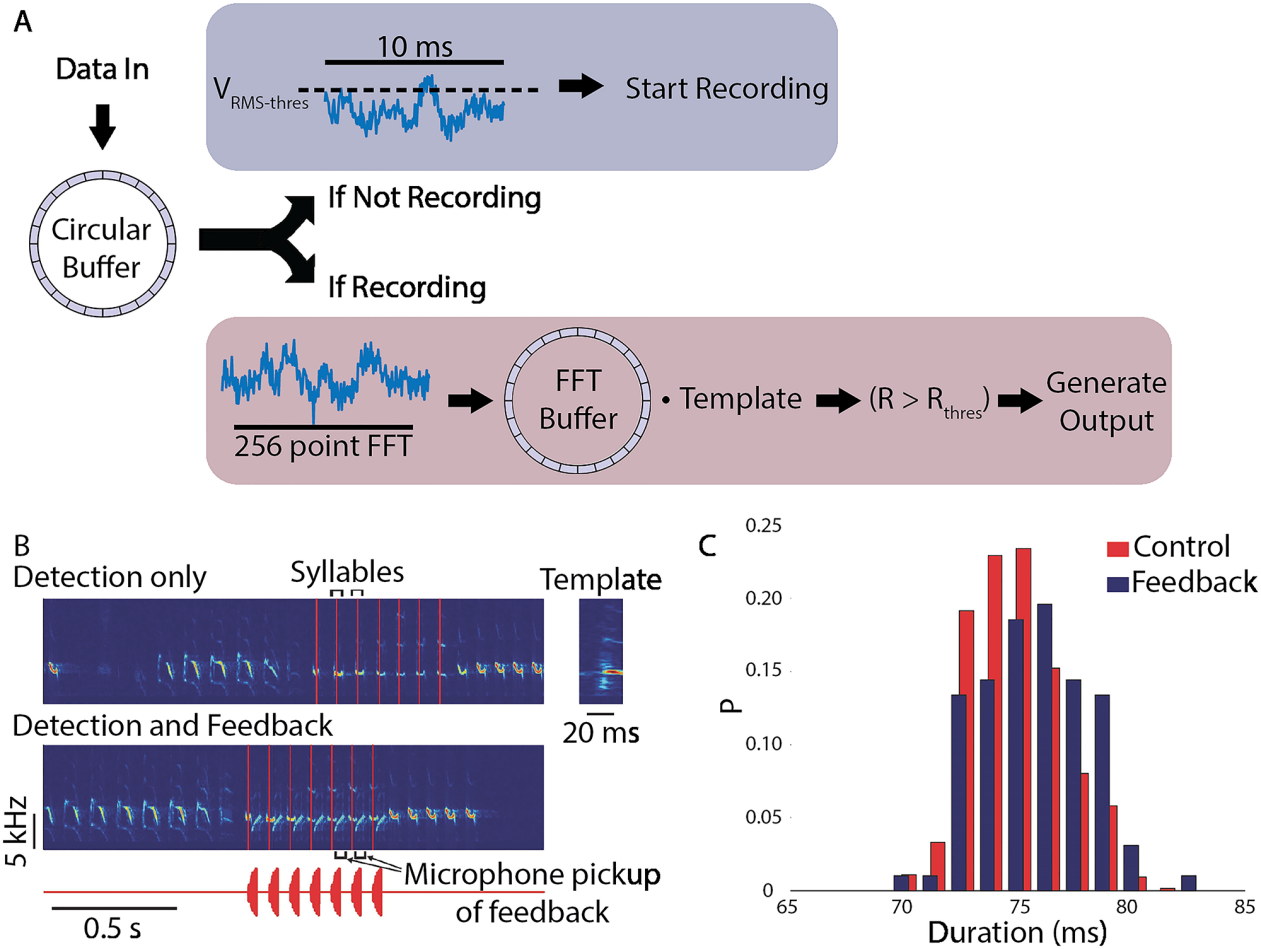
Hard RT distorted auditory feedback system. (A) Simplified diagram of the acoustic feedback system. When not triggered (top), the system computes the root mean square (RMS) of the input signal. When the RMS exceeds the threshold, the system is triggered. When triggered (bottom), the system computes the spectrogram of the most recent 20ms of signal and computes the correlation coefficient of this spectrogram with the spectrogram of the template sound (e.g., song syllable). The template sound is detected when the correlation coefficient exceeds a threshold value; in this case, acoustic feedback can be generated. Both the input and the acoustic output are saved to the computer hard drive. (B) Spectrogram of the song of a Bengalese finch and the times of occurrence of one of the song syllables. The system was programmed to only detect the occurrences of the target syllable in real time, no acoustic feedback was generated. The detection times are shown as vertical red lines. Bottom: the system is detecting the target syllables (vertical red lines) and is generating acoustic feedback after detection. The acoustic feedback waveform is shown below. The feedback signal is one of the birdsong syllables; the acoustic feedback pickup by the microphone is visible on the spectrogram. The zoomed-in spectrogram of the template is shown on the right. (C) DAF increases the duration of the time interval between Bengalese finch song syllables. Histogram depicts the time intervals between two subsequent syllables in the song in the presence of DAF (blue) and without DAF (red). The means are: Δ*t*_*mean*_ = 74.8ms (control, *N* = 637 syllables) and Δ*t*_*mean*_ = 75.7ms (feedback, *N* = 97 syllables), the difference is statistically significant (*p* = 0.001, two-way Kolmogorov—Smirnov test). All figures obtained and modified with permission from [38].

Skocik and Kozhevinokov [38] capitalized on the strengths of RTXI and modified it for experimental use in RT acoustic signal processing and feedback. Acoustic measurements were made by interfacing a microphone and amplifier to the analog inputs present on a National Instruments PCIe-6251 DAQ with a system frequency of 30 kHz. Acoustic feedback was generated by custom RTXI modules and delivered via the analog outputs on the DAQ. To minimize processing latency, the Data Recorder module was customized to provide two high-speed modes of operations—triggered (active) and non-triggered (idle). The complete process flow for the customized Data Recorder is shown in Fig 7. A circular buffer is inserted into the Data Recorder’s processing loop to enable buffering of acquired acoustic signals. The root mean square (RMS) is continuously quantified for the last 10ms of acquire data at a rate of 1 kHz. When the RMS exceeds the user-specified threshold, the system automatically switches to triggered mode operation.

Performance was also evaluated in experiments to detect specific syllables in the song of Bengalese finches. The finch song consists of a sequence of syllables separated by silences (inter-syllable gaps) (Fig 7), with variability introduced by the specific sequence of syllables in each song. Experiments were conducted to detect specific target syllables, with successful crossing of the correlation coefficient resulting in generation of acoustic feedback (either white noise or the song syllable). Fig 7B provides sample data demonstrating RTXI’s performance in both open-loop (detection only) and closed-loop modes (detection and feedback). System performance was characterized by comparing online syllable detection to off-line detection using spectrogram-based techniques. Over 92% of the target syllables (n = 659) were correctly identified by RTXI, with zero false positives. The undetected 8% are believed to be due to natural variability in acoustic structure that are not accounted for by the detection algorithm. Additional tests in Zebra finches resulted in a detection rate up to 96% (n = 756), with less than 1% false positives. In a final round of experiments, detection of target syllables was followed by DAF for modulation of timing between song syllables. Without DAF, the mean interval is 74.8 ms (n = 637), while with DAF the mean interval increases to 75.7 ms (n = 97).

These observations are consistent with previously published observations on the effects of RT feedback on song structure [39]. Moreover, they demonstrate the robust hard-RT capabilities of RTXI and the advantages of open-source software. Using RTXI enabled the authors to set up interfaces with different I/O hardware and modify the RTXI source code to run custom RT protocols.

#### Use case 3: EEG feedback-controlled transcranial alternating current stimulation

In addition to *in vitro* and *in vivo* closed-loop hard RT electrophysiology, RTXI’s flexibility, customizability, and hard RT performance has been utilized to investigate the effects of feedback-driven tACS. Brain stimulation using transcranial Alternating Current Stimulation (tACS) has gained significant momentum as an alternative to pharmacological methods for treatment of neurological and psychiatric disorders. By electrically stimulating the brain, aberrant network dynamics can be targeted with potentially higher efficacy, increasing therapeutic efficacy while minimizing undesired off-target effects [40, 41]. Unfortunately, most clinical investigations using tACS are conducted in a feedforward, open-loop manner. Given the success of feedback (closed-loop) control in a variety of engineering and neuroscience applications, Boyle et al [14] investigated the ability of electroencephalography (EEG)-measurement driven delivery of tACS to provide better control of cerebral cortex dynamics, which is implicated in several psychiatric illnesses.

tACS applies a weak sinusoidal electrical current to the scalp, resulting in a change in polarization of a large number of neurons, thus effectively altering neuronal network activity. Evidence from previous studies suggests that sinusoidal stimulation waveforms can be used to selectively modulate cortical oscillations at different frequencies commonly associated with different cognitive states. In their study, Boyle et al combined 40 Hz tACS with EEG measurements to control visual cortex state dynamics and modulate high- and low-alpha states induced by opening and closing the eyes.

**Fig 8:**
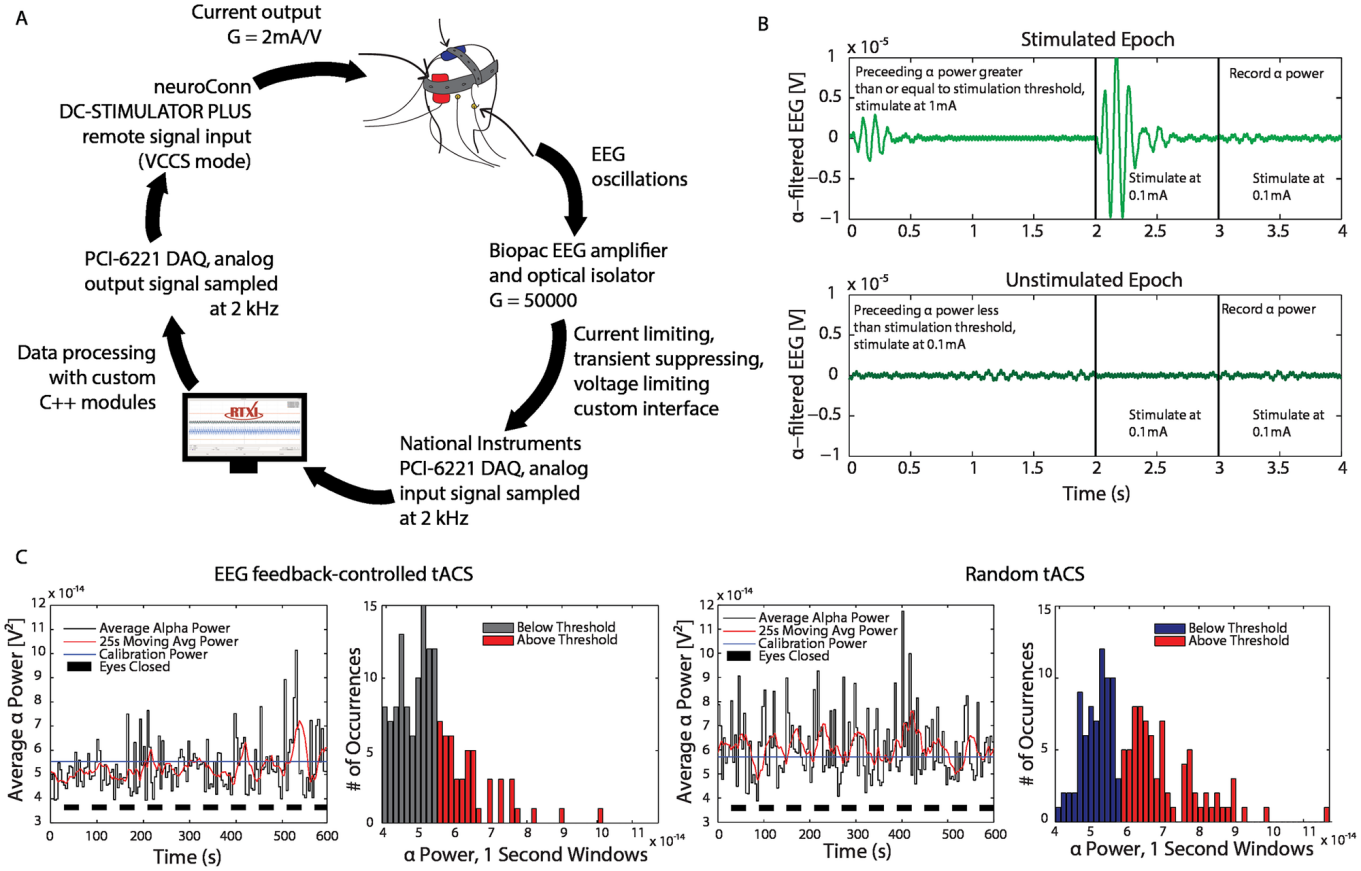
Optimization of therapeutic benefit of tACS via closed-loop EEG feedback-controlled tACS. (A) Hard real-time closed-loop protocol used for feedback-controlled delivery of tACS. EEG are measured and amplified prior to sampling with RTXI while the subject opens and closes their eyes every 30 seconds. EEG data from international 10–20 system sites O2, A1, and A2 are processed in hard RT using a custom module written to measure the alpha band power within a one second window. The computed power is validated against a threshold criteria, which determines the tACS amplitude to be delivered. The output from RTXI is connected to an external current-controlled stimulation and isolation unit. Sample alpha-filtered EEG traces with and without feedback-controlled tACS are shown in (B). Feedback-controlled tACS (C) almost completely suppressed alpha band power in a targeted way (Ratio EC_*α*_ to EO_*α*_ = 1.03, *p* = 0.041). Dose-matched random tACS (D) also suppressed alpha band power (normalized random-No Stim = −0.31, *p* = 0.0183), but was less effective than feedback-controlled tACS (normalized feedback_*α*_—random_*α*_ = −0.10, *p* = 0.0145). All figures obtained and modified with permission from [14].

RTXI was used in this investigation to measure and process incoming EEG activity, compute the appropriate output stimulation waveform in RT, and control timing of the experimental protocol. EEG measurements were made by interfacing with a Biopac EEG amplifier connected to a National Instruments PCI-6221 DAQ with a system frequency of 2 kHz. Measured EEG activity was processed online with custom C++ modules. Stimulation timing and waveforms were generated by RTXI and delivered to an isolation unit through the DAQ’s analog output channel. Fig 8A depicts the custom closed-loop, RT protocol engineered for this investigation.

All data processing and analysis was conducted in RT with custom modules written in C++. The incoming EEG data was filtered with a 6^th^ order Butterworth band-pass filter with a bandwidth of 8–12 Hz to isolate the alpha band. Power was then computed for the alpha band as the mean oscillation power with a 1 second window width. During the calibration period in each experiment, the module built a distribution with the computed alpha power values to determine the mean alpha power for both eyes-open and eyes-closed conditions. Stimulation thresholds were set to 1.05 of the average of the two mean alpha power values. The protocol then continued to assess mean alpha power at a rate of 0.25 Hz and modulated the stimulation depending on the measured alpha power. If alpha power was higher than the stimulation threshold, tACS was delivered to the subject for the first 2 seconds of the next epoch, otherwise stimulation was turned off. Each subject completed a total of four 12 minute recording sessions. Each recording started with a 2 minute calibration period. For the first 60 seconds, the subjects were asked to relax and be still with their eyes open. For the next 60 seconds, the subjects were asked to relax, be still, and close their eyes. After calibration, two recordings were conducted in each subject. First, subjects were asked to keep their eyes open (EO) and received EEG feedback-controlled tACS. For the second recording, subjects were asked to open and close their eyes when told by the experimenter every 30 seconds (EOEC) while receiving EEG-feedback driven tACS. These two recordings were repeated, however subjects received dose-matched randomly administered tACS.

The results of this investigation found that EEG feedback-controlled tACS can successfully suppress alpha power as well as state transitions caused by opening and closing of eyes. Subsequent investigations by the same group using RTXI have also demonstrated the ability of closed-loop tACS to boost sleep spindle activity and sleep-dependent motor memory consolidation [11]. These results demonstrate the customizability, modularity, and robust closed-loop RT performance of RTXI.

## Availability

RTXI is available under the GNU GPLv3 license and is publicly accessible online via GitHub (S3 File) along with over 50 custom modules contributed by the RTXI community. This repository enables tracking of user-submitted bugs and requests, and makes the continued development of RTXI publicly viewable. GitHub also allows users to watch the progress and development of RTXI, receive notifications as new updates or patches are submitted, and easily share custom modules built by any RTXI user. Users are encouraged to use this platform to communicate with the RTXI team with questions, comments, feature requests, and troubleshooting advice.

In addition to GitHub, a website (S3 File) is maintained and routinely updated with new troubleshooting tips and FAQs (S3 File) as well as a user manual and documentation (S3 File). Publications utilizing RTXI are indexed and listed for users to access key details associated with experimental setups, module use, and system parameters—enabling greater reproducibility of shared data. The RTXI team is dedicated to helping users with any aspect of RTXI—whether it is installation, creating custom modules, or integration of addition experimental hardware.

## Future directions

Advances in measurement and stimulation technologies over the last decade have increased both the spatial and temporal resolution at which scientists can investigate biological systems. Enhancements in micro-fabrication technologies have enabled development of high density silicon probes [42, 43], allowing large-scale spatial and temporal over-sampling of biological activity [44]. Development of ASICs has led to wide adoption of systems such as the low-cost and easy-to-use Intan headstages [45, 46]. Furthermore, recent developments in imaging technologies have made it possible to consider closed-loop applications in which the measured signal is an image representative of underlying electrical activity, rather than direct measurement of electrical potentials.

On-going development efforts within RTXI are focused on incorporating new measurement modalities (e.g., imaging) and acquiring from high channel count interfaces—all with hard RT closed-loop performance. For example, a image acquisition and processing module (GenICam) and a Ethernet-based data acquisition module (EthernetAcq) are now available for use within RTXI (S3 File). In many cases, hard RT closed-loop control with image or high channel count data processing can require more computation time than is available per cycle. On-going RTXI development efforts are also focused on providing API calls for distributing computational loads across dedicated processor cores and GPUs, with the goal of requiring little to no technical know-how on the user’s end.

The advent of optogenetics has enabled tight spatial and temporal control of stimulation of biological tissues [47–49]. These developments have spawned a significant interest in closed-loop control of biological systems [50], such as optical clamping of network activity [51] and optical shortening of cardiac action potential durations [52]. In most cases, delivery of optogenetic stimuli via a laser or diode is controlled and modulated by a voltage signal. For example, users can generate a standard stimulus waveform using the Signal Generator module or a custom stimulus waveform by providing an ASCII file to the Waveform Maker module and use it to control a laser or diode to achieve hard RT optogenetic stimulation. Such re-utilization of existing code and custom modules simplifies the transition to hard RT closed-loop control.

## Supporting information

S1 File Relevant terms and definitions used throughout the manuscript. (TEX)

S2 File Index of abbreviations used throughout the manuscript. (TEX)

S3 File Links for finding source code, tutorials, data, and user manuals for RTXI. (TEX)
