# Correction: Hard real-time closed-loop electrophysiology with the Real-Time eXperiment Interface (RTXI)

**DOI:** 10.1371/journal.pcbi.1005656

**Published:** 2017-07-17

**Authors:** The PLOS Computational Biology Staff

The initially published S1 File, S2 File and S3 File are in TEX format.

PDF copies of the Supporting Information are provided below.

## Supporting information

S1 File Relevant terms and definitions used throughout the manuscript. (PDF)

S2 File Index of abbreviations used throughout the manuscript. (PDF)

S3 File Links for finding source code, tutorials, data, and user manuals for RTXI. (PDF)
