# Supplementary material for: Correction: Hard real-time closed-loop electrophysiology with the Real-Time eXperiment Interface (RTXI)
Source: PLoS Comput Biol. 2017 Jul 17;13(7):e1005656. doi: 10.1371/journal.pcbi.1005656 (PMC5513696; doi:10.1371/journal.pcbi.1005656)
Supplement: S1 File — (PDF) [file pcbi.1005656.s001.pdf]

Supporting Information

S1 Terminology

- Hard real-time - guaranteed performance of I/O within specified bound; failure to achieve bounded performance results in a system crash
- Soft real-time - guaranteed performance of I/O within specified bounds; failure to achieve bounded performance is acceptable and does not effect system stability
- Open-loop - a process that computes an output based upon an input only (no feedback signal)
- Closed-loop - a process that computes an output based upon an input and feedback signal
- Kernel - core of an operating system that enables bi-directional communication between software and hardware
- Thread - a sequence of machine instructions for execution
- Scheduler - an operating system component that manages allocation of system resources
- Synchronous - unbuffered data acquisition or processing
- Asynchronous - buffered data acquisition or processing
- Jitter - deviation from the desired real-time period
- Real-time period - duration between the real-time thread waking up (becoming active) and going to sleep (becoming idle)
- Live CD - a complete bootable operating system with applications and drivers ready to run from a computer's memory; requires no installation
- Daemon - a computer process that runs as a background process (e.g., Dropbox, Skype but not Web Browsers or File Managers).
