# Supplementary material for: Correction: Hard real-time closed-loop electrophysiology with the Real-Time eXperiment Interface (RTXI)
Source: PLoS Comput Biol. 2017 Jul 17;13(7):e1005656. doi: 10.1371/journal.pcbi.1005656 (PMC5513696; doi:10.1371/journal.pcbi.1005656)
Supplement: S2 File — (PDF) [file pcbi.1005656.s002.pdf]

S2 Abbreviations

- RTXI - Real-Time eXperiment Interface
- RTOS - Real-Time Operating System
- GUI - Graphical User Interface
- I/O - Input/Output
- DAQ - Data Acquisition
- ADC - Analog-to-Digital Converter
- DAC - Digital-to-Analog Converter
- AI - Analog Input
- AO - Analog Output
- DIO - Digital Input/Output
- SISO - Single-Input Single-Output
- SIMO - Single-Input Multiple-Output
- MISO - Multiple-Input Single-Output
- MIMO - Multiple-Input Multiple-Output
- FLOPS - Floating-Point Operations per Second
- HDF - Hierarchical Data Format
- EEG - Electroencephalography
- tACS - Transcranial Alternating Current Stimulation
- ASIC - Application Specific Integrated Circuit
- CPU - Central Processing Unit
- GPU - Graphics Processing Unit
- RPM - Revolutions per minute
- GB - Gigabytes
- GHz - Gigahertz
- KHz - Kilohertz
- RAM - Random Access Memory
