# Supplementary material for: Correction: Hard real-time closed-loop electrophysiology with the Real-Time eXperiment Interface (RTXI)
Source: PLoS Comput Biol. 2017 Jul 17;13(7):e1005656. doi: 10.1371/journal.pcbi.1005656 (PMC5513696; doi:10.1371/journal.pcbi.1005656)
Supplement: S3 File — (PDF) [file pcbi.1005656.s003.pdf]

S3 Useful Links

A Website: https://rtxi.org

B Source code: https://github.com/RTXI/rtxi

C Compatible Hardware: http://rtxi.org/docs/manual/#daqs

D User manual: http://rtxi.org/docs/manual

E Tutorials and FAQs: http://rtxi.org/docs/

F User contributed modules: http://rtxi.org/modules/

G Support: http://rtxi.org/contact/

H Publications using RTXI: http://rtxi.org/papers/
